# Social and Environmental Impacts of Forest Management Certification in Indonesia

**DOI:** 10.1371/journal.pone.0129675

**Published:** 2015-07-01

**Authors:** Daniela A. Miteva, Colby J. Loucks, Subhrendu K. Pattanayak

**Affiliations:** 1 The Nature Conservancy, Fort Collins, CO, United States of America; 2 World Wildlife Fund-United States, Washington, DC, United States of America; 3 Duke University, Durham, NC, United States of America; Institute of Agronomy, University of Lisbon, PORTUGAL

## Abstract

In response to unsustainable timber production in tropical forest concessions, voluntary forest management certification programs such as the Forest Stewardship Council (FSC) have been introduced to improve environmental, social, and economic performance over existing management practices. However, despite the proliferation of forest certification over the past two decades, few studies have evaluated its effectiveness. Using temporally and spatially explicit village-level data on environmental and socio-economic indicators in Kalimantan (Indonesia), we evaluate the performance of the FSC-certified timber concessions compared to non-certified logging concessions. Employing triple difference matching estimators, we find that between 2000 and 2008 FSC reduced aggregate deforestation by 5 percentage points and the incidence of air pollution by 31%. It had no statistically significant impacts on fire incidence or core areas, but increased forest perforation by 4 km^2^ on average. In addition, we find that FSC reduced firewood dependence (by 33%), respiratory infections (by 32%) and malnutrition (by 1 person) on average. By conducting a rigorous statistical evaluation of FSC certification in a biodiversity hotspot such as Indonesia, we provide a reference point and offer methodological and data lessons that could aid the design of ongoing and future evaluations of a potentially critical conservation policy.

## Introduction

Tropical forests are of primary importance to biodiversity conservation and climate change mitigation [1,2]. However, in many locations they remain poorly protected and experience high levels of loss and degradation [3–5]. Timber production poses significant threats to forest ecosystems in tropical regions and Indonesia in particular [4,5]. For example, between 2000 and 2010 1.8Mha of forests (12.8% of all deforestation during that time period) were lost within commercial logging concessions in the country, with Kalimantan (the Indonesian part of the island of Borneo) being the most severely affected island [6]. Even selective logging can lead to degraded forests with low species richness [7].

Like in many other locations, establishing tenure in the form of forest logging concessions has often not been effective due to conflicting incentives and ineffective government responses [8,9]. In principle, traditional concessions employ the Indonesian Selective Cutting and Planting System (TPTI), which sets rotation time and the diameter of the harvested trees, necessitates replanting in the presence of low natural restocking rates and bans on logging on sensitive areas, but does not require reduced logging techniques [10]. However, the TPTI implementation has been flawed [10]. Furthermore, even when logging companies hold the formal rights over concessions, communities living in or around the concession land, especially those that are reliant on natural resources for their livelihoods, can also contribute to deforestation and degradation [11,12]. For example, in Berau District (East Kalimantan), about 150,000 cubic meters of timber (21% of all timber from the district) in 2000 were harvested by different illegal operations involving local communities [11]. These trends are likely to persist in Indonesia: Timber has become a significant source of revenue both for local governments and local communities because of the political and administrative decentralization process that began in 2001, coupled with the weak enforcement capabilities of the government [13,14]. Thus, because of mismanagement, deforestation and forest degradation due to uncontrolled logging and wildfires combined with the conversion of logged forests into oil palm plantations, the area of traditional logging concessions decreased from 59 million ha in 1990 to 25 million ha in 2011 [10].

In response to unsustainable practices in traditional logging concessions in Indonesia and elsewhere, companies have sought to adopt voluntary forest certification under which sustainable timber harvesting can become means to conserve forests and its species, while engaging and providing for local communities [9, 13–15]. Certification is based on the idea of creating incentives for sustainable forest management by providing a price premium or reputational benefits to the timber producers engaging in sustainable forest management as well as allowing access to markets that may be closed to timber producers with unsustainable forest practices [9,16].

**Fig 1:**
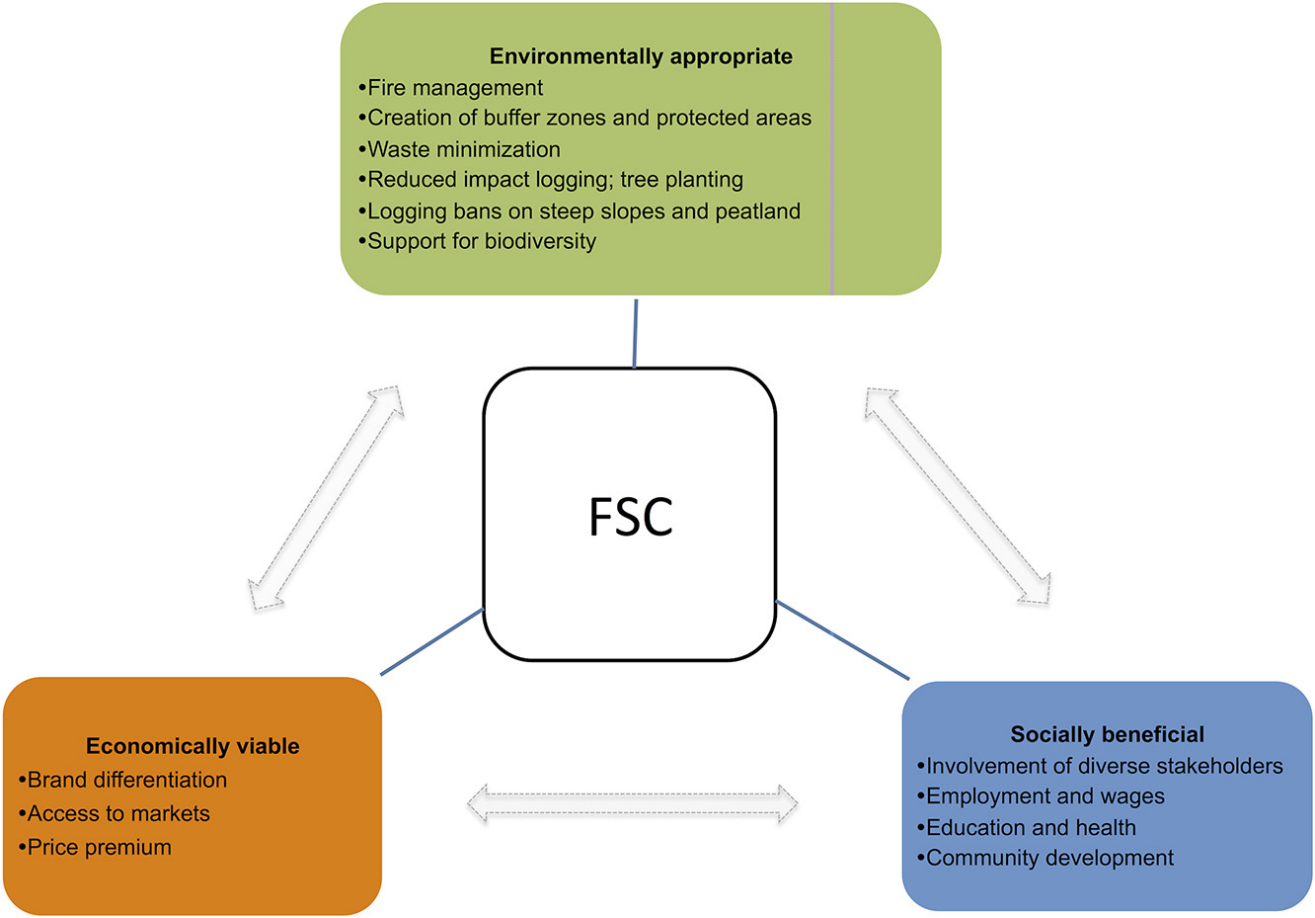
Summary of the intended FSC impacts. The arrows indicate linkages between the three goals. For example, reduced ambient pollution can reduce the incidence of diseases and hence household expenditures, increasing household welfare. The figure is based on [9,18,20].

We focus on evaluating the performance of the Forest Stewardship Council (FSC) forest management certification program in Kalimantan, Indonesia. Initiated by non-governmental conservation organizations, the FSC program emerged in 1993 to promote the sustainable management of forests globally [9]. The general goals of the FSC program are to achieve “environmentally appropriate”, “socially beneficial”, and “economically viable” timber harvesting in logging concessions [17]. In addition to providing incentives to companies to incorporate the social costs of timber production, the FSC certification criteria emphasize compliance with law and international agreements; tenure security and conflict resolution among stakeholders; recognition of indigenous people’s land rights; community relations and workers’ rights; investments to maintain biodiversity; the ecological productivity of the area; minimized waste and damage to other resources like soil and water due to road construction; enhanced forest regeneration, monitoring and assessments of impacts of activities; and maintenance of high conservation value forests [17] (Fig 1).

Our review of the published background papers and grey literature on Indonesian FSC [18–20] suggests that to promote environmentally responsible logging, the FSC concessions adopt reduced impact logging practices of selective logging and tree planting (*Tebang Pilih Tanaman* and/or *Tebang Pilih Tanaman Jalur*), improved fire management, winch-based reduced skidding, and waste minimization. In addition, the FSC concessions in the country often include riparian buffer zones, areas with steep slopes or peat, reserves and protected areas that are maintained by the concessionaires but cannot be logged. The social goals are achieved through the employment of local workers and appropriate compensation as well as by implementing programs to improve education, health care, infrastructure, and community development (*e*.*g*., *Pembinaan Masyarakat Desa Hutan*–Forest Village Development Program). The FSC operations in Indonesia also implement general programs and commit specific resources to local communities that are affected by FSC [18]

Despite the recent proliferation of tropical forest management certification in Indonesia and elsewhere [21], there is almost no causal evidence about their performance promoting sustainable forestry and development in the impacted areas [20,22,23]. Because FSC concessions are not randomly established, an increasingly popular way to assess causality is through rigorous quasi-experimental techniques that use a mix of baseline measurements and other observable features (often called covariates) for FSC to identify non-FSC ‘controls’. These controls stand in for what would have happened to a FSC plot, had it not been certified (also referred to a “counterfactual”) [20,24,25]. By controlling for issues like price trends and threat from deforestation, these quasi-experimental techniques lead to statistically rigorous evidence about the performance of a conservation intervention [20,24–26].

Very few studies have attempted to examine the causal impacts of the FSC program using a quasi-experimental approach. Brandt et al (2014) examine how the source of foreign capital (which presumably could influence the extent of compliance with sustainable forestry) impacts deforestation in the Congo basin, but do not focus on policies per se, including FSC certification [27]. Using data from community forest management associations in Acre, Brazil, de Lima et al (2008) to find some evidence that FSC increased awareness of regulations and management plans, better waste disposal, better use of fire, and restrictions on hunting [28]. Medjibe et al (2013) examine whether number of damaged trees, length of skid trails, width of logging roads, changes in species composition and loss in the above-ground-biomass is different on a FSC certified plot compared to adjacent conventional logging plot in Gabon [29]. Cerutti et al (2011) argue that FSC reduced harvested timber volumes by 18% in Cameroon [30]. However, the design of the latter two studies does not allow for statistically rigorous inference. Collectively, these studies confirm the lessons from recent reviews of better management practices for timber and for forest certification that very few studies use a statistically rigorous empirical design in general, focus on tropical forests and on Asia [20,23,31].

Following numerous calls for evaluating the performance of certification programs [20,23,32], we present the first statistically rigorous evaluation of the environmental and socio-economic impacts of FSC certification in Kalimantan. To our knowledge, this is one of very few studies filling the important knowledge gap regarding whether or not FSC helps preserves forests and improves the socio-economic dimensions of human wellbeing in a setting where it potentially matters most. Compared to our effort, most of the literature comprises of consulting reports with unknown scientific protocol for data collection and analysis, micro-ecological studies of limited scale (e.g., a few plots in idiosyncratic landscapes) and limited scope (e.g., ignore poverty and other socio-economic outcomes) or rhetorical policy opinion pieces. More generally, our work responds directly to recent reviews and calls for rigorous evaluation of conservation policies [26,33,34].

## Materials & Methods

### Time period and region

We focus on the impact of the FSC forest management certification program between 2000 and 2008 in Kalimantan (Fig 1, Table 1). A blend of practicality and innovation influenced the choice of data used in our analysis. For example, the spatial and temporal frames were determined by the data availability. Because only two FSC concessions were established outside Kalimantan (in Sulawesi and Sumatra) prior to 2008 and because there are no reliable spatial boundaries for the original (pre-2008) concessions in Sumatra and Sulawesi, we did not include them in our analysis. While some firms applied for FSC certification as early as 2000, most completed the process in 2006 (or very close to 2006). For this reason, we use 2006 to mark of the start of FSC certification for the concessions in our sample and treat the year 2000 as our baseline.

**Table 1:** List of the FSC certified plots in Kalimantan by 2008.

Concessioner	Year concession certified	Year concession allocated	Area certified (ha)	Primary Products	Ownership
PT. Intraca Wood Ind	2006	1988	194,250	Plywood	PT Inhutani I (24.7%), PT. Altracks ‘78 (49.5%), PT Berca Indonesia (24.7%), PTIM Employees Cooperative (0.96%)
PT. Sari Bumi Kusuma I Dan Ii (block Seruyan)	2007	1998	144,729	Plywood, Bankirai decking and moulding products	PT SBK (100%)
PT. Sumalindo Lestari Jaya Ii	2006	1981	257,793	Wood panels, plywood, solid wood, veneer	PT Sumber Graja Sejahtera (75%), PT Barito Pacific Timber (9.53%), general public (15%)
PT. Erna Djuliawati	2005	1999	180,489	Container flooring, truck flooring, plywood, fancy panels and door skins, engineered flooring	Lyman Grp (98%), 2% by 17 local cooperatives

The logging concessions (HPH) are allocated for 35–70 years. As of 2008, in Indonesia the forest management FSC certification spans about 4.1% of the forest area designated for logging.

### Unit of Analysis and Treatment Definition

**Fig 2:**
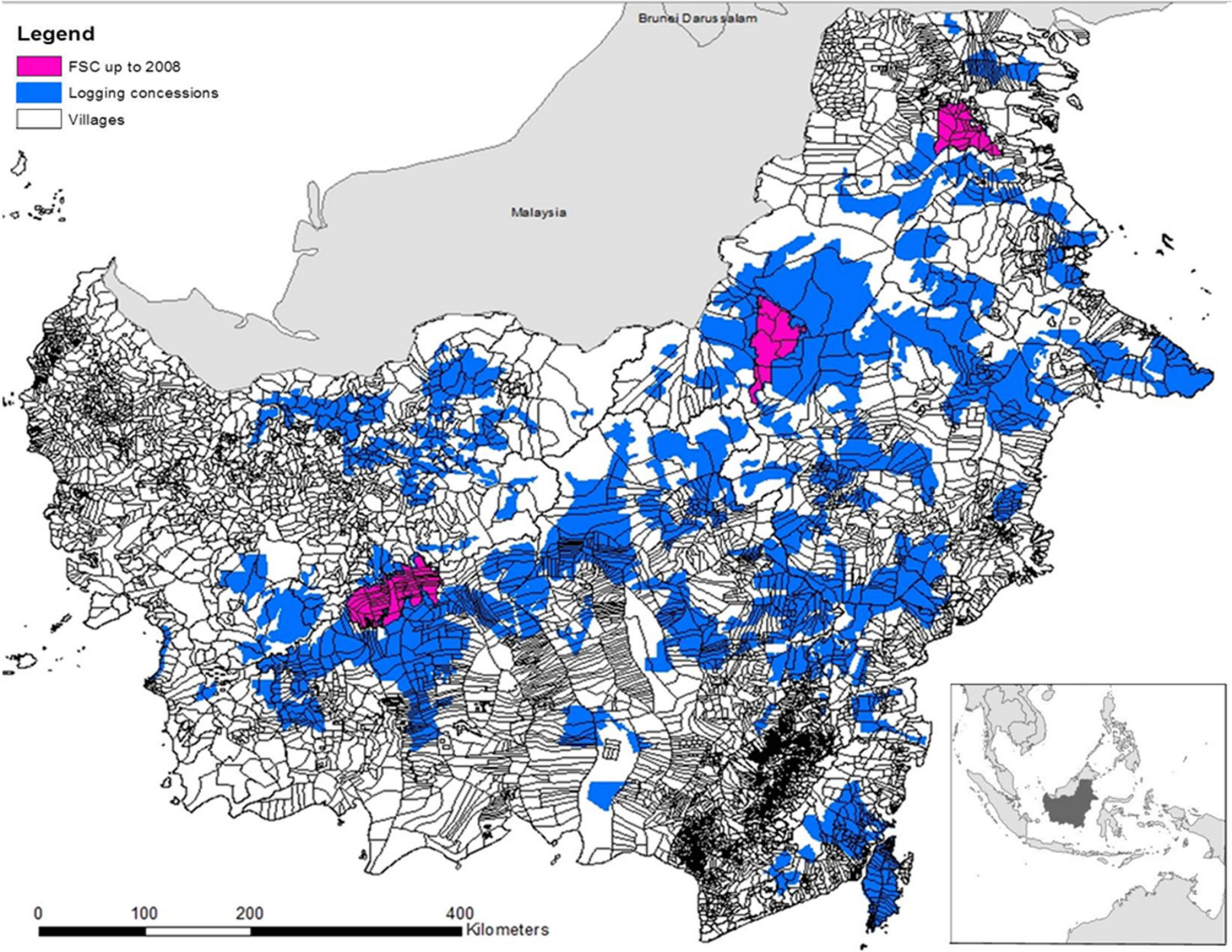
Distribution of the logging concessions in Kalimantan. The FSC concessions established prior to 2008 appear in pink. The dark blue polygons are all logging (HPH) concessions established by 2008. A village is considered treated if it overlaps with a FSC forest concession; similarly, a control village is one intersected by an HPH concession.

As the unit of analysis we use a village, which is combination of human settlements and adjacent land as mapped by the Indonesian census. There are three main reasons for this choice. First, as there is no unclaimed land, the village areas sit entirely within concessions area (Fig 2). Because FSC impacts forests through both reduced impact logging and the creation and support of protected areas important to the provision of clean water or biodiversity (Fig 1), our ecological indicators (e.g., deforestation and forest fragmentation) capture the cumulative impacts of any FSC activities at the village level. While a pixel-level analysis can also capture the impacts of reduced impact logging by matching observationally similar pixels within the production portions of FSC and traditional logging concessions, it is likely to miss the impacts of the forest protection activities of FSC concessions. Second, village-level characteristics (e.g. poverty levels and population density) are likely to affect the performance of a FSC certified concession. However, a pixel or another purely spatial unit does not have socio-economic-political characteristics. If we were to account for the socio-political and economic characteristics (see discussion of confounders below), we would have to either assign the same values to each pixel that falls within the same village or use some spatial statistical algorithm to interpolate them. Either procedure would raise concerns about auto-correlation of spatially proximal pixels, especially if there is a spatial weighting process for assigning socio-economic values to pixels. Third, since many of the goals of FSC target communities, a pixel has no socio-economic meaning.

The treated group comprises all villages in Kalimantan that partially or fully overlap with a FSC concession established prior to 2008 (Fig 2). As the control group, we use all villages inside or intersected by traditional logging (Hak Pengusahaan Hutan (HPH)) concessions in Kalimantan. The plots in the control villages were not certified under FSC or the Indonesia Ecolabel Institute (LEI), which is an Indonesian standard consistent with FSC, by 2008 [35]. Field studies and annual audits conducted by the Rainforest Alliance are the primary source of details on the practices within logging concessions for our study area [16]. Some external verification comes from a different study on only a subset of the concession plots we consider in this paper (e.g., [19].

### Covariates

We focus on the village characteristics that are likely to influence both (a) the placement of FSC concessions and (b) the outcomes. Following the growing literature on quasi-experimental methods, our selected covariates include population density, poverty levels, the length of the river network within a village as rivers can be used for transporting the harvested timber, the type of property rights (private ownership and customary land ownership proxied by the village area under a property rights regime divided by the village area) within a village, proximity to markets and mills, slope, elevation, baseline forest cover, peat land area, and village area falling under Protected Areas (Table 2). The proximity to markets is proxied by distance to major cities, distance to province capitals, distance to the nearest market with permanent structures, and the distance to the nearest port interacted with the sea depth at the port, in order to distinguish between ports based on their commercial importance (larger vessels necessitate deeper ports). These are consistent with previous recommendations [20]. All of the covariates refer to the year 2000 and, therefore, to the period prior to certification. Descriptive statistics for the covariates are reported in S2 Table.

**Table 2:** Definitions of the covariates used in the analysis.

Variable (Variable codes in parentheses)	Definitions
Average slope	Average slope within a village, in degrees
Average elevation	Average elevation for a village, in meters
Fraction village under protection	Fraction village area under any designated protected area
Distance to major city	Euclidean distance from the village boundary to the nearest district capital or major trading centers, in meters
Distance to capital	Euclidean distance from the village boundary to the nearest province capital, in meters
Distance to ports*depth of port (dist2ports*depth)	Euclidean distance from the village boundary to nearest port (in km)*sea depth at the port (in km).
Length of the river	Length of the river network within a village, in meters
Distance to permanent markets	Proximity to the nearest market with permanent structures. Based on PODES, in km.
Population density	#people/village area. Based on PODES
Poverty rate	#Poor households/#Total households within a village
Distance to mills	Euclidean distance to the nearest processing mill, in meters
Fraction village area under peat	Unitless
Fraction village area under customary ownership	Unitless. Calculated as area under customary ownership/total village area as reported by the Indonesian Village Census
Fraction village area under private ownership	Unitless. Calculated as area under private ownership/total village area as reported by the Indonesian Village Census.
Village area (vil_area)	In hectares

### Outcomes

The performance of FSC certification in terms of achieving environmental and social goals is the focus of this paper. We evaluate the impact of FSC on three sets of indicators: (1) Environmental: change in the amount and distribution of forest cover, incidence of forest fires, air and water pollution; (2) Household welfare: fuelwood dependence, incidence and levels of malnourishment, the incidence of acute respiratory infections (ARI); and (3) Village development: village infrastructure and the availability of funding for the village. Detailed descriptions of the outcomes are presented in Table 3. The multiple period data (2000, 2006 and 2008) allow us to compare absolute changes as well as the rates of change pre- and post-certification.

**Table 3:** Definitions of the main outcomes.

Outcome	Definitions
Average forest cover	Average % forest within a village, range:0–100; the dataset aims to exclude plantations and vegetation less than 5 meters in height. Source: MODIS
Malnutrition	#Individuals in 2008 suffering from malnutrition in the past 3 years. Calculated per village. Source: PODES
Cumulative #Forest fires 2000–2008 on forest cells ≥40% forest	This is the #total village fires occurring on forest cells with >40% forest. Because the number of fires within a year varies depending on anthropogenic activity as well as on phenomena like El Nino and La Nina, we calculated the cumulative number of fires within the period. We experimented with different definitions of “forest fires”: fires that occur on cells with specified % forest cover (10% or 40%), fires that fall within villages with at least some percent forest (10% or 40%). We also standardize the counts using the villages areas under forest (using the 10 or 40% cutoff). These results are very similar to the ones included in the paper and are available upon request. Source: NASA FIRMS
Air pollution	These are self-reported measures of whether or not air pollution was present in the village in a particular year (1 if air pollution present 0 otherwise). Source: PODES
Water pollution	These are self-reported measures of whether or not water pollution was present in the village in a particular year (1 if water pollution present 0 otherwise). Source: PODES
Firewood	Indicates whether the village relies on firewood as the main source of fuel (1 if firewood is primary, 0 otherwise). Source: PODES
Availability of street lights in a village	1 if available, 0 otherwise. Source: PODES
Integrated Health Centers (IHC)	Number of integrated health center facilities within a given year. Source: PODES
Incidence of Acute Respiratory Infections (ARI)	1 if there is at least 1 recorded incidence of ARIs within a village and 0 otherwise. Source: PODES
Availability of private funding	1 if funding from non-government domestic sources available in 2008; 0 otherwise. Source: PODES

The choice of environmental indicators directly follows from the FSC goals (Fig 1). We focus on the extent of habitat (forest cover), its structure (perforated area, core area) and indicators of environmental quality (forest fires, air pollution, water pollution). We would have preferred to have indicators of ecosystem function (e.g., the provision of hydrological services, pollination) or biodiversity. Unfortunately, wall-to-wall data on such indicators are non-existent at a fine resolution for most parts of the world, including our study site. The choice of our indicators for social and economic outcomes also balances the practicality of what is available with what is conceptually correlated with these outcomes. For example, we include three well measured and, therefore, frequently used indicators of socio-economic wellbeing, including the numbers of people in a community who: (i) suffer from malnutrition, (ii) suffer for acute respiration, and (iii) rely on fuelwood, which is a cheaper and dirtier cooking fuel in Indonesia [36]. Similarly, we also include indicators of village development and infrastructure (street lights, health posts and private finance) that best match the descriptions of the community programs in the Kalimantan FSC concessionaires [18].

While FSC in Kalimantan can be characterized as a complex coupled socio-ecological system, we chose these indicators also because they matched the FSC goals. For example, the FSC environmental goals of managing fires, creating buffer zones and protected areas, preventing logging on steep slopes, adopting reduced impact logging and tree planting could all reduce forest cover loss, while changing habitat structure. Similarly, the FSC economic goals of obtaining price premiums and market access, when combined with social goals of providing employment and wages, improving education and health, and conducting community development–could all collectively change household and community level wellbeing, proxied by the indicators listed above. These could be direct or indirect to economic or environmental channels. Although there may be questions about the reliability of some of these proxy indicators, if our proxy is riddled with noise, then the noise would dominate the signal and we should see no impact of the policy. Thus, even though our estimates are conservative at best, we are not overstating the impacts of the program.

### Data sources

#### Socio-economic data

The socio-economic data used in the analysis come from the PODES datasets, which are village-level censuses carried out by the Indonesian government every 3 years to collect data on land use, population demographics, and village infrastructure for all nearly 69,000 villages in Indonesia. The village-level information is collected from the village leader (kepala desa or lurah, in rural and urban areas, respectively) by representatives of the Indonesian Bureau of Statistics [37,38]. Using a unique village identifier, we construct a panel consisting of the 2000, 2006, and 2008 PODES data. We use data from 2000 as our baseline as some FSC concession holders started the pre-certification compliance procedures shortly after. The latest PODES data we have are from 2008.

#### Forest cover data

The forest cover data come from the MODIS Vegetation Continuous Fields datasets [39]. These datasets provide estimates of the percent tree cover within each cell at 250 m resolution for each year between 2000 and 2010. The main text presents the results for the change in forest cover between 2000 and 2008; the results using 2010 as the final year are presented in S4 Table.

#### Fire data

We use data on the location of active fires using the NASA FIRMS datasets [40]. The datasets give the temporal and spatial distribution of active fires for the whole globe. We classified the fires into forest vs. non-forest fire categories based on the previous year’s land cover pixel on which the fire point was located. This was necessary because of the annual level of aggregation of the forest cover data: Because we can only assign a forest cover value to a pixel for a given year and not for specific month/day, it is not possible to distinguish whether the fire event on a particular pixel preceded the measurement of the forest cover for that pixel within a given year. We used cumulative counts of the number of forest fires, between 2000 and 2008, in order to avoid bias associated with the choice of baselines because of the high inter-annual variability of fire incidence in Indonesia.

#### Physiographic data

Data on the proximity to cities and ports, slope and elevation were collected from the World Resource Institute and the University of Georgia GIS data repository [41,42]. All distances are based on the Euclidean distance from the village edge to a city (port, mill) center.

#### Fragmentation data

We conducted fragmentation analysis to assess the configuration of the landscape in terms of core and perforated habitat because these are important proxies for disturbance and species diversity and richness [43]. In order to calculate the fragmentation statistics for our study area, we reclassified the MODIS forest data using a 70% forest cover as the threshold defining forest. We chose 70% as the cutoff as the resulting forest area most closely resembled the previous estimates of forest area in 2000 [44]. Thus, cells with more than 70% forest were classified as forest and the rest as non-forest. We performed the analysis using a version of the Landscape Fragmentation Tool [45,46].

The tool classifies the forested pixels into 4 main categories: (1) “Edge”- forest pixels along the exterior perimeter of a forest that are often associated with higher tree mortality, high predation and introduced species (these are referred to as “edge effects”) [47]; (2) “Patch”, which comprises small isolated fragments of forest that are often degraded by edge effect; (3) “Perforated”-forest pixels along the edge of an interior gap in a forest that may be degraded by edge effects. In our study area these are likely the effect of selective logging; and (4) “Core”, which includes interior forest pixels that are likely unaffected by edge effects (see S2 Fig for an illustration of the output). Because of the resolution of the original MODIS datasets, we used an edge distance of 250m.

### Methods

FSC certification is a voluntary process. Historically, FSC plots have been established in areas with relatively low population density, higher poverty and in forests with more valuable timber [32,48]. To control for the endogenous (non-random) placement of the certification program, we apply matching methods that allow us to use existing data on non-certified villages, in order to infer what would have happened in villages overlapping FSC concessions, if FSC certification had not been introduced there [25]. The goal of the matching procedure is for each FSC village to find an observationally similar (in multivariate space) village within a traditional logging concession. Specifically, we use a Mahalanobis distance metric as our indicator of multivariate space and a nearest neighbor matching with replacement procedure [49]. The advantage of distance metric is that it gives more weight to covariates with smaller variances and smaller weights to noisier covariates. The procedure identifies the best match to a treated village based on the smallest distance between the treated village and a control village [49]. The causal impact of the program, the Average Treatment Effect on the Treated (ATT), is calculated as the average of the differences between the outcome for the treated village and the outcome for the matched control for each matched pair.

To improve covariate balance, we also apply trimming based on an estimated propensity score, which is the predicted linearized probability of FSC certification estimated in a logit model [50]. The logit model includes factors that (1) affect the placement of the FSC concessions and the outcomes, (2) have not been affected by the intervention, and (3) do not create misspecification issues like collinearity [51]. The actual variables used in this estimation are reported in S2 Table. The procedure allows us to ensure that the matches have a common support and eliminate observations with extreme values of the propensity score. We combine this trimming with matching based on the Mahalanobis nearest neighbor approach because it yielded the best covariate balance and the smallest remaining bias compared to a propensity score matching or a covariate matching using a Mahalanobis distance metric and no trimming. Post-matching, we apply linear bias adjustments to the ATT to remove any remaining differences in the covariate distributions [52–54]. We also correct the standard errors for heteroskedasticity within a treatment arm [53,54].

For the majority of outcomes we conduct difference-in-difference matching. Specifically, because we have data for most outcomes for three years (2000, 2006, and 2008) that correspond to time before certification, the year of certification, and time after certification, we apply triple-difference estimation. This approach uses as the outcome the difference between the differences in the outcomes between 2000 and 2006 and the differences in the outcomes between 2006 and 2008. Specifically, the outcome for each group is constructed as (y_2000_-y_2006_)-(y_2006_-y_2008_), where y_200X_ is the outcome for a given year. In other words, the approach compares the change in the outcome before certification to change in the outcome after certification for the treated and control villages.

The treatment effect for each matched pair is given by

$${\left[(\text{y}2000-\text{y}2006)-(\text{y}2006–\text{y}2008)]\right|}_{\text{treated}}–{\left[(\text{y}2000–\text{y}2006)–(\text{y}2006–\text{y}2008)]\right|}_{\text{control}}$$

Thus, a positive ATT implies that the rate of change for the outcome for the treated group exceeds the rate of change of the outcome for the control group. In other words, if the outcome is a desirable one (e.g., % forest cover), a positive value tells us that, compared to before FSC certification, the introduction of certification led to a faster rate of forest cover increase in the treatment group compared to the matched controls. Note that in contrast to a simple difference-in-difference estimation, the ATT depends on the relative change of the outcomes between periods. In effect, we estimate a net reduction in deforestation through this procedure. For example, the raw data suggests that both FSC and non-FSC concessions experienced deforestation between 2000 and 2006, but that the FSC concessions showed faster recovery post-treatment compared to non-FSC concessions. The main advantage of our approach is that, while controlling for the observed characteristics, it allows us to eliminate unobserved time trends and not just time-invariant linear characteristics (eliminated by the more popular double difference estimators) that may be systematically different for the two treatment groups [55].

For a few of the development indicators, only cross-sectional data (data for a single year) were available. For those we employed cross-sectional matching, such that the treatment effect for a matched pair is y2008_treated_ – y2008_control_, where y2008 is the outcome for 2008. Positive values in this case indicate that FSC increased the values of the outcome in the treated villages compared to the matched control villages.

## Results

The descriptive statistics of the covariates for the sample prior to matching (S1 Table) and the estimation of the propensity score used for trimming (S2 Table) identify some spatial features of FSC villages (*i*.*e*., those spanned by FSC concessions). Relative to villages within traditional logging concessions, the FSC villages are located in isolated areas with lower population density, higher poverty rates, and closer to mills. Furthermore, they tend to have less peatland and shorter river networks (thus, fewer opportunities to transport illegal harvests). The non-random placement of the intervention justifies the application of a quasi-experimental technique like matching [52,56].

**Fig 3:**
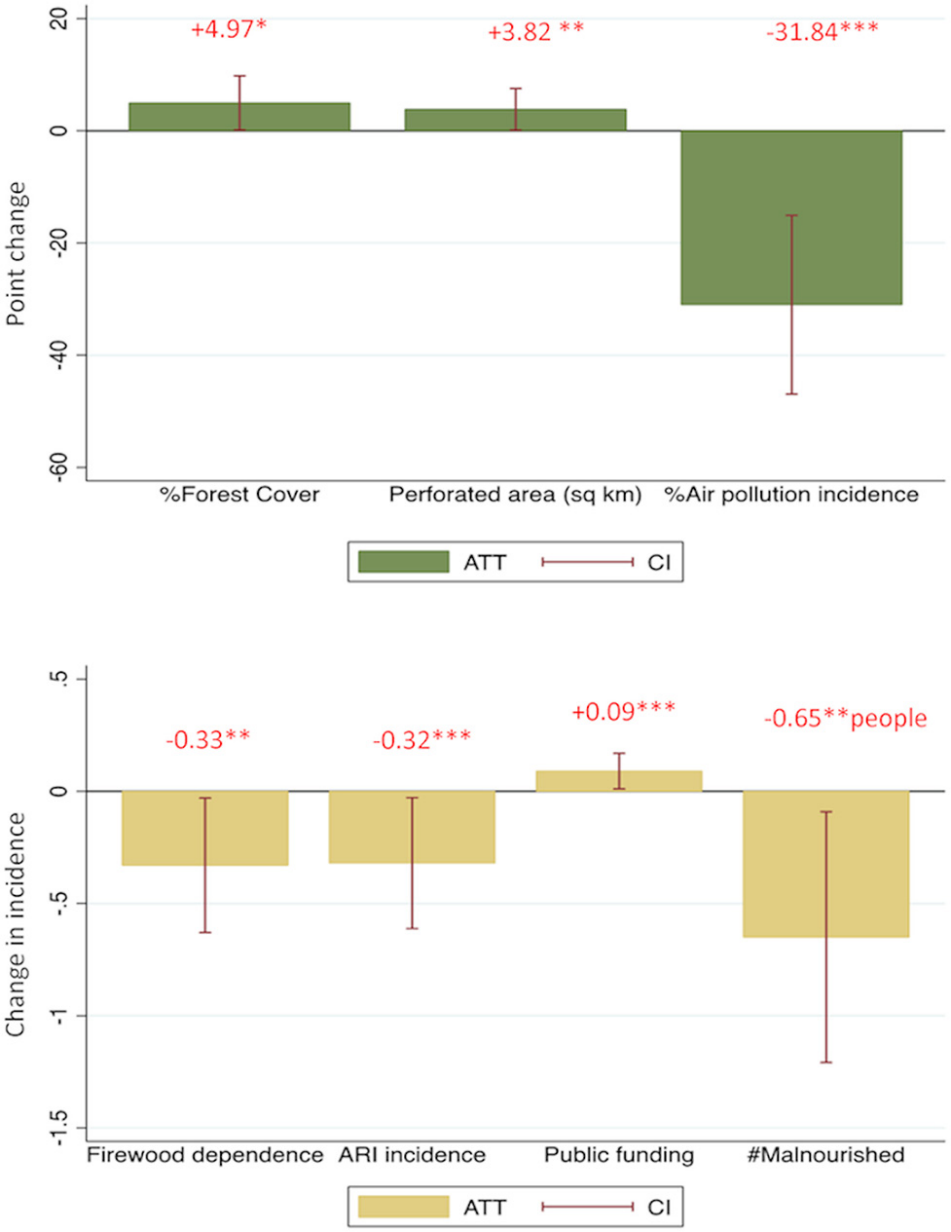
Social and Ecological Impacts of FSC in Kalimantan. The heights of the columns represent the size of the impact; only statistically significant changes in the environmental (top) and socio-economic (bottom) outcomes are presented (if the error bars do not cross 0 (the x-axis), it suggests the impact was statistically significantly different from 0 (= no impact)). The impact of the program (ATT) is given in red above each bar. Significance levels: ***1%, **5%, *10%.

Fig 3 summarizes the statistically significant outcomes. In Table 4, we report our findings (ATT estimates) from the matching strategy on various outcomes. Note, the results are based on nearest neighbor matching using (a) Mahalanobis distance metric, (b) post-matching bias and variance correction, and (c) triple differences. We find that compared to villages in uncertified logging concessions, FSC certification in Kalimantan increased forest cover by about 5 percentage points in certified villages.

**Table 4:** Estimated treatment effects on the treated (ATT) for the selected environmental and socio-economic outcomes (heteroskedasticity-corrected standard errors (se) in parentheses) N_t_, N_cm_, and N_c_ refer to the number of matched treated, matched control, and the control pool, respectively.

Outcome	Mean treated	Mean controls	Bias Adj. ATT	N_t_/N_cm_/ N_c_
Avg percent forest cover change (2000–2008) (3D)			4.97*	
	19.99	15.02	(2.90)	67/33/832
Change in air pollution incidence b/w 2000 and 2008 (3D)			-0.31***	
	-0.12	0.19	(0.06)	66/32/796
Change in water pollution incidence between 2000 and 2008 (3D)			-0.10	
	0.12	0.22	(0.11)	66/32/796
Cumulative #Forest fires 2000–2008 on forest cells ≥40% forest			-6.26	
	8.04	14.30	(6.93)	67/33/832
Perforated area (3D), in sq m			3,822,164.0**	
	4,159,382.5	337,218.5	(1,861,812.1)	67,33, 832
Core area (3D), in sq m			-3,680,405.0	
	54,452,542.2	58,132,947.2	(13,286,917.0)	67,33, 832
Change in firewood dependence 2000–2008 (3D)			-0.33**	
	0.00	0.31	(0.15)	66/32/796
Change in ARI incidence 2000–2008 (3D)			-0.32***	
	-0.12	0.20	(0.11)	66/32/796
# Malnourished in 2008			-0.65**	
	0.30	0.94	(0.28)	67/33/832
Change in main street lights 2000–2008 (3D)			-0.03	
	-0.11	-0.07	(0.09)	66/32/796
Change in the #IHC 2000–2008 (3D)			0.17	
	0.42	0.26	(0.23)	66/32/796
Private funding available in 2008			0.09***	
	0.09	0.00	(0.03)	67/33/832

Triple difference estimators are given as (3D). We provide robustness checks for the environmental outcomes using data for 2000–2010 in S4 Table.

Significance levels

***-1%

**-5%.

*10%

FSC also increased perforated areas. Collectively, these forest cover outcomes are indicative of selective logging within the certified concessions. We also find evidence that FSC reduced the incidence of air pollution within the treated villages, but did not have a statistically significant impact on the incidence of fires between 2000 and 2008. One potential explanation for the cleaner air but unchanged incidence of fires is that FSC reduced the intensity and emissions from fires, while having no statistically significant impact on fire incidence, which is a binary indicator and, thus, less statistically powerful. However, our data do not allow us to test this hypothesis.

Our results indicate that FSC certification also impacted household well-being. Compared to the villages within non-certified logging concessions, FSC reduced fuelwood dependence, the incidence of acute respiratory illness (ARI) and of malnutrition in 2008 (Table 4). Compared to the villages within non-certified concessions, FSC increased the village funding from private sources in 2008, consistent with the suggestions in the gray literature [18]. However, there is no statistically significant impact on the rates of providing infrastructure such as streetlights and health centers. A likely explanation is that infrastructure takes time to put in place.

Potential alternative explanations to the observed patterns could be that FSC induced differential migration, with poor sick forest-dependent people leaving the certified villages. If such people moved out of the FSC villages, we would observe increased household welfare and decreased forest loss. Alternatively, the observed forest cover patterns could also be attributed to FSC shifting the agricultural practices within a village away from swidden (ladang) cultivation. Despite our attempt to assemble a comprehensive data rich in many dimensions, we cannot rule out these happenstances. However, we can test for differences between FSC and control villages for some of these indicators. We fail to reject the null hypotheses that there are statistically significant differences in the rate of population change (ATT = -378.18, se = 378.97) or in the fraction of village land under agriculture (ATT = -0.13, se = 0.20). Thus, we did not find support that differential migration or decreases in ladang are driving our results.

## Discussion

Our paper adds to the slowly growing literature on conservation policy impacts of forest certification. First, our example from Kalimantan employs several best practices in impact evaluations to reduce the bias in our estimate and strengthen our claim that we have measured the causal impacts of FSC certification. Second, while much of the literature has focused on protected areas, payments for environmental services or forest decentralization mostly in Latin America (e.g., Brazil, Costa Rica and Mexico), we are the first to consider certification–a potentially important tool for forest conservation–in a global deforestation hotspot such as Indonesia [23,33]. Finally, unlike the usual focus on aggregate deforestation and poverty outcomes, we respond to recent calls to better tailor impact evaluations by examining a variety of outcomes such as forest fragmentation, fire, air pollution, and community welfare, in addition to deforestation.

Using data from 2000–2008 in Kalimantan, we find that FSC certification significantly reduced deforestation by 5 percentage points and air pollution by 31% compared to the matched control villages in non-certified logging concessions. This suggests that the program may address previous concerns about the ineffectiveness of traditional logging concessions in reducing deforestation and their potential to decrease community welfare by limiting access to forest resources [9]. However, while FSC certification improves some environmental and socio-economic outcomes, the program may introduce disturbances in forest ecosystems (e.g., by opening the canopy).

It is remains unclear if the FSC certification of logging concessions promotes biodiversity per se. Previous studies have found that sustainably managed forest concessions represent a “middle ground” between pristine habitats and logged areas [32], with the selectively logged forests in Kalimantan retaining a substantial fraction of the original species richness and diversity [57–61]. However, studies also document differential impact on species guilds. For example, Edwards et al (2009) found that forest rehabilitation of selectively logged forests in Kalimantan was beneficial to insectivorous birds, but detrimental to frugivores [58]. Mammal species are generally found to persist in selectively logged forests, despite lower abundances [60]. These studies suggest the impact of FSC certification will likely depend on the conservation goal (a few target species vs. species richness). Similarly, because FSC does not specifically target carbon in its guidance for certification or forest management requirements, in some cases it may not have significant impacts on reducing carbon emissions [19]. The impacts of FSC on the provision of other ecosystem services also remain unknown.

Because the sustainability of the FSC certification largely depends on the involvement of the local communities, it is important the program generates benefits to them [20]. We find that, compared to the villages in non-certified logging concessions, FSC in Kalimantan generated positive benefits to local communities (e.g., reduced disease incidence and fuelwood dependence, and increased private funding). However, the data do not allow us to examine the mechanisms through which FSC effects change among villages. Establishing the mechanisms for change are important next steps in the design and implementation of FSC and conservation programs in general [33]. Further, we likely need more time to detect if FSC leads to noticeably better household and community outcomes (e.g., better public infrastructure), compared to the more sensitive and immediate environmental outcomes, especially for some of the more recent concessions (e.g., PT. Sari Bumi Kusuma). Despite being a relatively large sample study spanning multiple years, we do not have the luxury of studying only the older concessions. Thus, our estimates should be viewed as conservative estimates of early socio-economic impacts of FSC.

Owing to hurdles in obtaining certification and to the lack of explicit consideration of biodiversity and ecosystem service provision, FSC may not be an effective conservation or emissions mitigation policy by itself. However, because of the compatibility of REDD + and FSC requirements, FSC certification can be used as the stepping stone to receiving carbon payments that can be used to subsidize certification and make it accessible to more firms [22,32,62]. Another way to increase the effectiveness of FSC in preserving habitats may entail combining protected areas with FSC concessions [63] or with another intervention, especially in a way that promotes spatial coordination among targeted forests as opposed to the current concession-level planning for FSC forest management [32].

These conclusions lead us to re-emphasize the paucity of causal evidence of the performance of FSC certification and similar programs, despite their recent proliferation in tropical and non-tropical regions [64]. Maintaining the credibility of the FSC program necessitates rigorous empirical evaluations [20,65]. In contrast to the deforestation metrics that employ geospatial data, the socio-economic indicators of program performance and proxies for the impacts on biodiversity and ecosystem services are not readily available and necessitate on the ground surveys by independent third parties. Unfortunately, the current design of the FSC program does not require detailed data collection [20,65]. For this reason, we call for structural changes that make more and better data collection an integral part of the program implementation; this would facilitate evaluation of their effectiveness and long-term impacts, and, thus, improve conservation outcomes. In the meanwhile, we hope that the concepts, methods and data presented in this paper can expand and diversify the toolkit for evaluating a potentially important conservation policy, even as it serves as a point of reference and offers lessons for improving future research on forest certification, such as the coordinated effort by CIFOR currently underway [20].

## Supporting Information

S1 Appendix Supplementary Information. The file contains additional robustness checks and background information.
(DOCX)

S1 Fig Spatial distribution of the matched treated (n = 67) and matched control villages (n = 33). (TIFF)

S2 Fig Illustration of the output from GUIDOS/Landscape Fragmentation Tool. In the analysis, we collapsed the three types of core areas into a single category.
(TIFF)

S1 Table Descriptive statistics for the covariates used for matching (statistics reported for the unmatched sample). Absolute values for the normalized differences greater than 0.25 flag different covariate distributions [52].
(PDF)

S2 Table Results from the first stage of the matching (logit model to generate the propensity scores). The dependent variable is whether or not a village falls under an FSC concession. Because of multicollinearity, the average % forest cover in 2000 was dropped from the analysis.
(PDF)

S3 Table Covariate balance tests for the sample using only uncertified logging villages as controls. (PDF)

S4 Table Results for the rate of change between 2000 and 2010 (triple differences). Standard errors are given in parentheses. Similar to the results for the period spanning 2000 and 2008, the outcome variable for each of the treatment arms is defined as (outcome 2000-outcome 2006)-(outcome2006-outcome2010). All the areas are defined in terms of square meters. A positive value indicates that FSC increased outcome (e.g., forest cover) in the treated villages relative to observationally similar non-FSC villages.
(PDF)

S5 Table Changes in forest cover (triple differences) excluding protected zones. Standard errors are given in parentheses and the t-statistics-in brackets. Similar to previous results, the outcome variable for each of the treatment arms is defined as (outcome 2000-outcome 2006)-(outcome2006-outcome _end year_), where the end year is either 2008 or 2010. A positive value indicates that FSC increased forest cover in the treated villages relative to observationally similar non-FSC villages.
(PDF)

S6 Table Coordinates of the centroids of the concessions included in the study. (PDF)
