# Supplementary material for: Social and Environmental Impacts of Forest Management Certification in Indonesia
Source: PLoS One. 2015 Jul 1;10(7):e0129675. doi: 10.1371/journal.pone.0129675 (PMC4488465; doi:10.1371/journal.pone.0129675)
Supplement: S1 Appendix — The file contains additional robustness checks and background information. (DOCX) [file pone.0129675.s001.docx]

# Supporting Information

# Social and Environmental Impacts of Forest Management Certification in Indonesia

## FSC certification in Indonesia

Certification is done at the concession plot level, is voluntary for logging firms, and is performed by private entities. In Indonesia FSC certification is carried out by the Rainforest Alliance Smartwood program. The certification process involves review of the documentation, site visits, and interviews with local stakeholders including communities living in/around the concession plot, with audits taking place annually. When a firm fails to comply with FSC regulations, it is issued a Corrective Action Requirement (CAR). If a major CAR is not met within the specified timeframes, the FSC certification can be temporarily suspended. More details on the performance for each concession are available here on the Rainforest Alliance website, which is the third-party certifying organization in Indonesia [1]. The primary cut control requirements are area-based [1].

The FSC principles are incompatible with conversion concessions (HTI) issued after 1994. They also preclude logging in riparian buffers, biodiversity reserves, in protection forest buffer zone and forests on land with slope greater than 40%. Because of the very few observations located on such slopes, we could not empirically test whether FSC certification reduced logging there.

The coordinates for all concession plots included in our sample are presented in Table S6.

### Indonesian alternatives to FSC

A local timber certification program, the Sustainable Natural Production Forest Management (SNPFM), also exists and is carried out by the Indonesia Ecolabel Institute (LEI). The two certification schemes have consistent criteria and certification requirements [2,3]. The four FSC concessions in our sample were also certified under LEI. Because Sarmiento Parakanca Timber (SARPATIM) obtained LEI certification in mid December 2008 and FSC in 2011 [4], we retain the villages overlapped by the concession in the control group. As a result, we expect that our estimates would be biased towards 0 (i.e. finding a smaller impact of FSC). The rest of the plots that did not obtain FSC certification before 2008, had not obtained LEI certification by that date, either. In this paper we will focus on the impact of FSC certification, with the caveat that it significantly overlaps with LEI. We do not aim to compare the effectiveness of one certification scheme over another.

## Additional analysis

### Triple difference analysis using 2000-2010 data

On order to address concerns about the short post-FSC period our data span, we consider environmental outcomes (forest amount and configuration) until 2010 in the statistical procedure described in the main text. The results are presented in the Appendix (Table S4-S5). The results indicate that FSC stalled deforestation rates by about 9% compared to non-certified concession villages (Table S4). As the magnitude of these impacts is considerably higher than for 2000-2008, we expect that FSC effectiveness will continue to increase in the future.

We find evidence that FSC may increase rate of edge areas (Table S4). This is consistent with continued logging. While the results are suggestive that FSC many reduce forest fires, the results were not statistically significant.

### Total aggregate forest impacts vs. changes in the annual rates of deforestation

Note that our metrics for deforestation and forest fragmentation do not measure the annual rates of forest cover change. While the latter is an interesting statistic, it has not been used in the literature on conservation impact evaluation, to the best of our knowledge. We could compute the annual rates of forest change for each period using the formula: [(new value/old value)^(1/#years)]-1 before and after certification for the FSC and non-FSC control villages [5]. The bias adjusted ATTs are -.03 (suggesting FSC increased the annual forest cover by 3%), which is statistically significant at the 1%, and -.01 (or 1% increase in forest cover), which is not statistically significant at the 10%, for the 2000-2010 and 2000-2008 periods, respectively. Qualitatively, the annual rates are consistent with the estimates based on percentage points.

### Evaluating impacts on forest cover in the logging zones of the concessions

The logging concessions in our sample sometimes overlap with protected areas. Thus, the %forest cover outcome reported in the main paper captures the impact of FSC both in the logging zones and within protected areas. In order to examine only the impact of FSC within the non-protected zones of the logging concessions, we repeated the analysis excluding protected areas. The results are consistent with those reported in the main paper, suggesting that FSC also improved logging practices (Table S5).

## Bibliography

1. Rainforest Alliance (2014) Certified forestry operation summaries for Indonesia. Available: http://www.rainforest-alliance.org/forestry/certification/transparency/operation-summaries-idn.

2. LEI (2013) Memorandum of understanding LEI-FSC. Available: http://www.lei.or.id/mou-between-fsc-and-lei; http://www.lei.or.id/comparability-study-of-lei-and-fsc.

3. Auld G, Gulbrandsen LH, McDermott CL (2008) Certification schemes and the impacts on forests and forestry. Annu Rev Environ Resour 33: 187–211. Available: http://dx.doi.org/10.1146/annurev.environ.33.013007.103754.

4. Tropical Forest Foundation (2012) PT Sarmiento Parakantja Timber. Available: http://www.tff-indonesia.org/index.php/en/ril-a-certification-newsletter/2410-pt-sarmiento-parakantja-timber-certified.

5. FAO (1995) Forest Resources Assessment 1990. Global Synthesis. Rome.
