# Supplementary figures and images for: Social and Environmental Impacts of Forest Management Certification in Indonesia
Source: PLoS One. 2015 Jul 1;10(7):e0129675. doi: 10.1371/journal.pone.0129675 (PMC4488465; doi:10.1371/journal.pone.0129675)

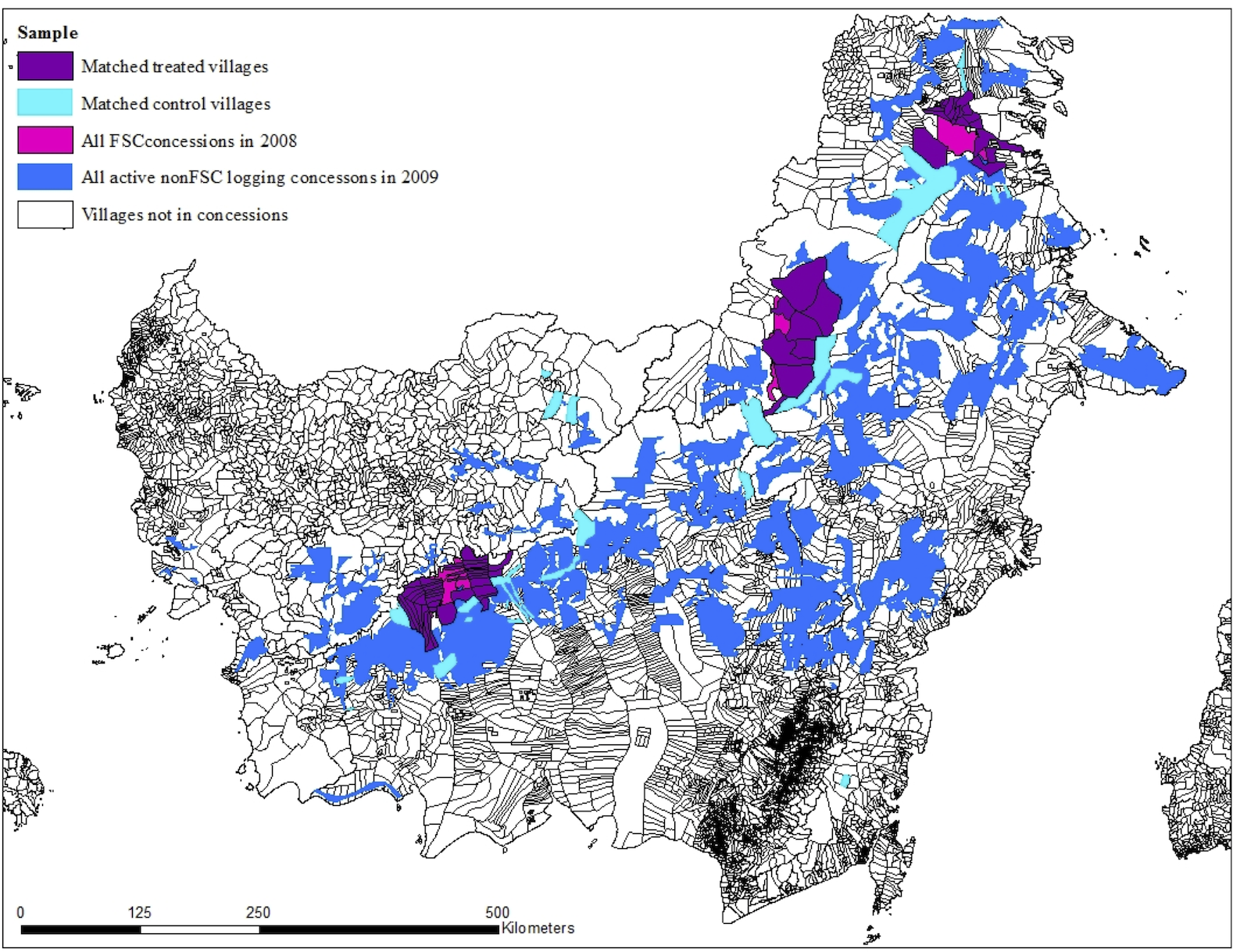

Supplement: S1 Fig — (TIFF) [file pone.0129675.s002.tiff]

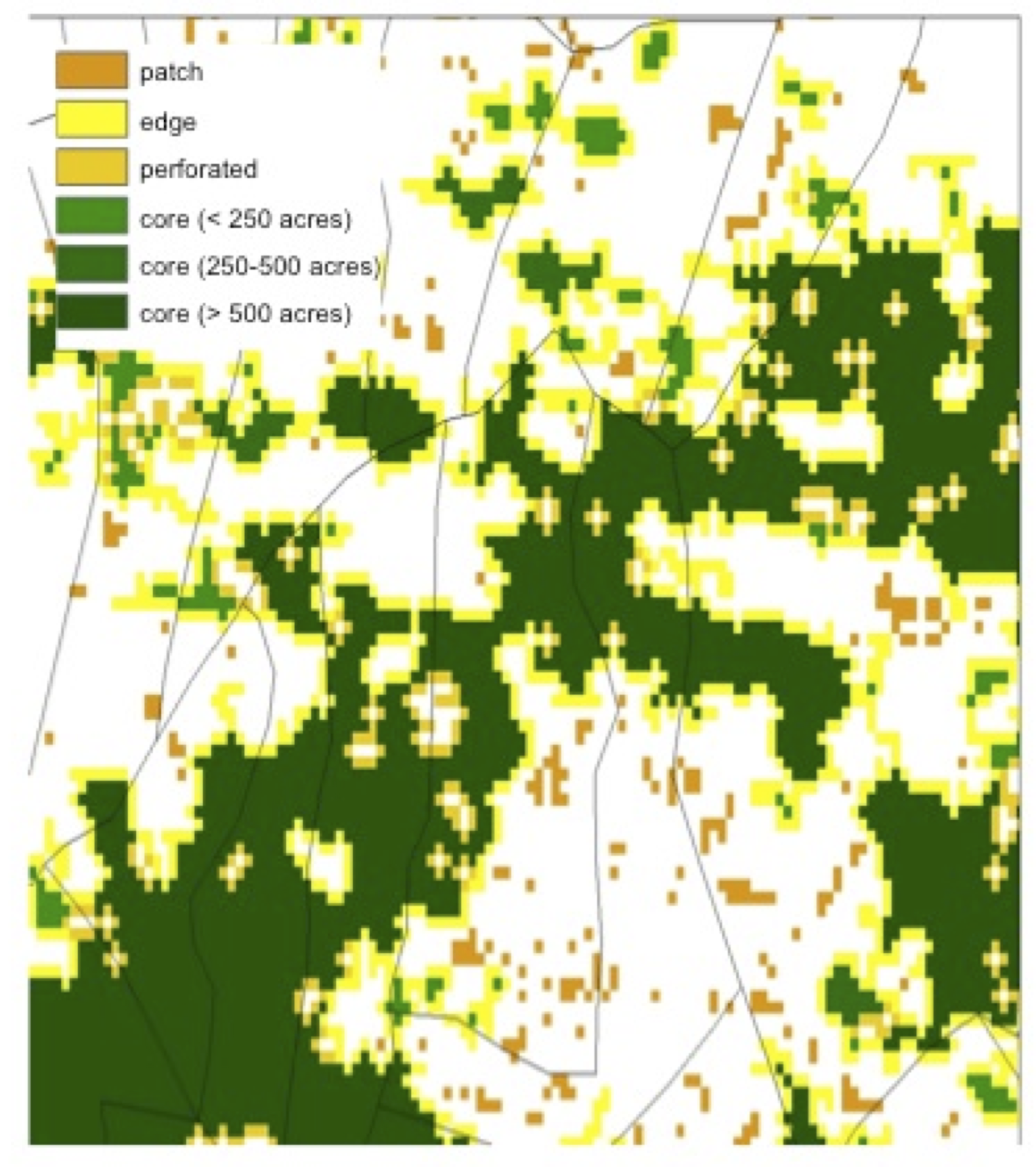

Supplement: S2 Fig — In the analysis, we collapsed the three types of core areas into a single category. (TIFF) [file pone.0129675.s003.tiff]
