# Supplementary material for: Social and Environmental Impacts of Forest Management Certification in Indonesia
Source: PLoS One. 2015 Jul 1;10(7):e0129675. doi: 10.1371/journal.pone.0129675 (PMC4488465; doi:10.1371/journal.pone.0129675)
Supplement: S1 Table — Absolute values for the normalized differences greater than 0.25 flag different covariate distributions [52]. (PDF) [file pone.0129675.s004.pdf]

Covariates	Control villages N	Mean	St. Dev	Villages under FSC N	Mean	St. Dev	Norm diffs
Average forest cover in 2000	1160	53.79	15.56	76	68.25	6.60	0.86
Average slope (degrees)	1160	5.36	4.03	76	8.09	3.37	0.52
Fraction under protected areas (fr_pa)	1160	0.05	0.16	76	0.02	0.12	-0.12
Distance to city (meters)	1160	60556.61	40022.77	76	85061.63	57496.47	0.35
Distance to province capital (meters)	1160	216551.50	88676.02	76	301015.00	75746.74	0.72
Distance to ports*depth	1143	0.36	0.32	73	0.29	0.20	-0.19
Length of the river network (meters)	1160	21504.47	35108.33	76	25046.68	39657.65	0.07
Distance to permanent markets (km)	1100	41.24	36.13	76	76.92	36.96	0.69
Average elevation	1160	150.73	167.89	76	279.57	242.72	0.44
Population density	1159	32.93	143.42	76	4.20	7.42	-0.20
Poverty rate in 2000	1159	0.56	0.29	76	0.72	0.27	0.40
Distance to mills in 2004 (meters)	1160	60480.30	41381.82	76	61656.82	23927.94	0.02
Fraction peatland	1160	0.05	0.17	76	0.02	0.08	-0.20
Fraction land under customary ownership (adat)	884	0.00	0.01	73	0.00	0.01	0.05
Fraction land under private property	884	0.18	0.26	73	0.19	0.20	0.03
Village area (ha)	1160	179.75	330.10	76	231.24	354.56	0.11
