# Supplementary material for: Social and Environmental Impacts of Forest Management Certification in Indonesia
Source: PLoS One. 2015 Jul 1;10(7):e0129675. doi: 10.1371/journal.pone.0129675 (PMC4488465; doi:10.1371/journal.pone.0129675)
Supplement: S2 Table — The dependent variable is whether or not a village falls under an FSC concession. Because of multicollinearity, the average % forest cover in 2000 was dropped from the analysis. (PDF) [file pone.0129675.s005.pdf]

Covariate	Only logging villages as controls
Distance to city (sq root) , in m	6.63E-04<br>(2.60E-03)
Distance to ports*distance to city, in m	8.03E-11***<br>(1.59E-11)
Distance to province capital, in m	1.83E-05***<br>(2.43E-06)
Distance to ports*depth at port (in m*ft)	-0.20<br>(0.78)
Length of the river network (^-4), in m	-0.15***<br>(0.03)
Distance to permanent markets, in m	0.03***<br>(0.01)
Average elevation, in m	-0.02<br>(0.03)
Population density (^-4)	-1.58***<br>(0.48)
Poverty rate in 2000	1.70***<br>(0.54)
Distance to mills, sq root	-4.64E-03**<br>(2.16E-03)
Fraction village land with peat	-7.76***<br>(1.73)
Constant	-4.70***<br>(1.57)
n	1155
LR chi2(10)	197.27
P	<0.0001

Significance levels: ***-1%, **-5%., *-10%
