# Supplementary material for: Social and Environmental Impacts of Forest Management Certification in Indonesia
Source: PLoS One. 2015 Jul 1;10(7):e0129675. doi: 10.1371/journal.pone.0129675 (PMC4488465; doi:10.1371/journal.pone.0129675)
Supplement: S3 Table — (PDF) [file pone.0129675.s006.pdf]

<table>
<tr><th></th><th></th><th colspan="2">Mean</th><th></th><th></th><th colspan="2">t-test</th><th></th></tr>
<tr><th>Variable</th><th>Sample</th><th>Treated</th><th>Control</th><th>%bias</th><th>%reduct |bias|</th><th>t</th><th>p>|t|</th><th>norm diffs</th></tr>
<tr><td rowspan="2">Average %forest in 2000</td><td>Unmatched</td><td>68.49</td><td>42.46</td><td>196.8</td><td></td><td>12.02</td><td>0.00</td><td></td></tr>
<tr><td>Matched</td><td>68.49</td><td>68.13</td><td>2.7</td><td>98.6</td><td>0.38</td><td>0.70</td><td>0.02</td></tr>
<tr><td rowspan="2">Average slope</td><td>Unmatched</td><td>7.95</td><td>3.42</td><td>126.4</td><td></td><td>9.7</td><td>0.00</td><td></td></tr>
<tr><td>Matched</td><td>7.95</td><td>8.04</td><td>-2.5</td><td>98</td><td>-0.15</td><td>0.88</td><td>-0.02</td></tr>
<tr><td rowspan="2">Fraction under protected area</td><td>Unmatched</td><td>0.02</td><td>0.04</td><td>-13.2</td><td></td><td>-0.93</td><td>0.35</td><td></td></tr>
<tr><td>Matched</td><td>0.02</td><td>0.04</td><td>-8.1</td><td>38.8</td><td>-0.53</td><td>0.60</td><td>-0.06</td></tr>
<tr><td rowspan="2">Distance to major city</td><td>Unmatched</td><td>80141.00</td><td>36161.00</td><td>99.4</td><td></td><td>10.55</td><td>0.00</td><td></td></tr>
<tr><td>Matched</td><td>80141.00</td><td>84564.00</td><td>-10</td><td>89.9</td><td>-0.58</td><td>0.56</td><td>-0.07</td></tr>
<tr><td rowspan="2">Distance to province capital</td><td>Unmatched</td><td>3.00E+05</td><td>1.50E+05</td><td>161.4</td><td></td><td>11.59</td><td>0.00</td><td></td></tr>
<tr><td>Matched</td><td>3.00E+05</td><td>3.00E+05</td><td>8.9</td><td>94.5</td><td>0.57</td><td>0.57</td><td>0.06</td></tr>
<tr><td rowspan="2">Distance to ports*depth of port</td><td>Unmatched</td><td>0.29</td><td>0.29</td><td>-2.2</td><td></td><td>-0.17</td><td>0.87</td><td></td></tr>
<tr><td>Matched</td><td>0.29</td><td>0.28</td><td>2.6</td><td>-17.5</td><td>0.16</td><td>0.87</td><td>0.02</td></tr>
<tr><td rowspan="2">Length of the river network within a village</td><td>Unmatched</td><td>24036.00</td><td>11124.00</td><td>39.6</td><td></td><td>4.17</td><td>0.00</td><td></td></tr>
<tr><td>Matched</td><td>24036.00</td><td>23760.00</td><td>0.8</td><td>97.9</td><td>0.04</td><td>0.97</td><td>0.01</td></tr>
<tr><td rowspan="2">Distance to permanent markets in 2000</td><td>Unmatched</td><td>75.51</td><td>24.02</td><td>151.6</td><td></td><td>13.89</td><td>0.00</td><td></td></tr>
<tr><td>Matched</td><td>75.51</td><td>75.51</td><td>0</td><td>100</td><td>0</td><td>1.00</td><td>0.00</td></tr>
<tr><td rowspan="2">Average elevation</td><td>Unmatched</td><td>257.83</td><td>86.79</td><td>88.4</td><td></td><td>8.67</td><td>0.00</td><td></td></tr>
<tr><td>Matched</td><td>257.83</td><td>234.09</td><td>12.3</td><td>86.1</td><td>0.69</td><td>0.49</td><td>0.09</td></tr>
<tr><td rowspan="2">Population density in 2000</td><td>Unmatched</td><td>4.33</td><td>337.61</td><td>-29.5</td><td></td><td>-1.71</td><td>0.09</td><td></td></tr>
<tr><td>Matched</td><td>4.33</td><td>3.19</td><td>0.1</td><td>99.7</td><td>1.06</td><td>0.29</td><td>0.00</td></tr>
<tr><td rowspan="2">Poverty rate in 2000</td><td>Unmatched</td><td>0.71</td><td>0.47</td><td>86.6</td><td></td><td>7.06</td><td>0.00</td><td></td></tr>
<tr><td>Matched</td><td>0.71</td><td>0.75</td><td>-15.9</td><td>81.6</td><td>-0.87</td><td>0.39</td><td>-0.11</td></tr>
<tr><td rowspan="2">Distance to mills</td><td>Unmatched</td><td>61070.00</td><td>45725.00</td><td>51.2</td><td></td><td>3.61</td><td>0.00</td><td></td></tr>
<tr><td>Matched</td><td>61070.00</td><td>59214.00</td><td>6.2</td><td>87.9</td><td>0.38</td><td>0.70</td><td>0.04</td></tr>
<tr><td rowspan="2">Fraction under peatland</td><td>Unmatched</td><td>0.02</td><td>0.12</td><td>-51.2</td><td></td><td>-3.13</td><td>0.00</td><td></td></tr>
<tr><td>Matched</td><td>0.02</td><td>0.02</td><td>2.2</td><td>95.7</td><td>0.28</td><td>0.78</td><td>0.02</td></tr>
<tr><td rowspan="2">Fraction under adat in 2000</td><td>Unmatched</td><td>0.00</td><td>0.00</td><td>2.3</td><td></td><td>0.14</td><td>0.89</td><td></td></tr>
<tr><td>Matched</td><td>0.00</td><td>0.00</td><td>4.9</td><td>-115.2</td><td>0.77</td><td>0.44</td><td>0.03</td></tr>
<tr><td rowspan="2">Fraction private land in 2000</td><td>Unmatched</td><td>0.18</td><td>0.41</td><td>-73.2</td><td></td><td>-4.73</td><td>0.00</td><td></td></tr>
<tr><td>Matched</td><td>0.18</td><td>0.14</td><td>12.2</td><td>83.4</td><td>1.14</td><td>0.26</td><td>0.09</td></tr>
<tr><td rowspan="2">Village area</td><td>Unmatched</td><td>218.56</td><td>87.47</td><td>47.5</td><td></td><td>5.03</td><td>0.00</td><td></td></tr>
<tr><td>Matched</td><td>218.56</td><td>226.46</td><td>-2.9</td><td>94</td><td>-0.14</td><td>0.89</td><td>-0.02</td></tr>
</table>
