# Supplementary material for: Social and Environmental Impacts of Forest Management Certification in Indonesia
Source: PLoS One. 2015 Jul 1;10(7):e0129675. doi: 10.1371/journal.pone.0129675 (PMC4488465; doi:10.1371/journal.pone.0129675)
Supplement: S4 Table — Standard errors are given in parentheses. Similar to the results for the period spanning 2000 and 2008, the outcome variable for each of the treatment arms is defined as (outcome 2000-outcome 2006)-(outcome2006-outcome2010). All the areas are defined in terms of square meters. A positive value indicates that FSC increased outcome (e.g., forest cover) in the treated villages relative to observationally similar non-FSC villages. (PDF) [file pone.0129675.s007.pdf]

Outcome	Mean treated	Control	Bias adj ATT
Average % forest cover (3D)	35.95	27.28	8.67***<br>(2.83)
Patch area (3D), in sq m	-4,744,054.43	-6,645,413.43	1,901,359.00<br>(1,238,082.26)
Edge area (3D), in sq m	6,543,607.44	-11,556,392.56	18,100,000.00***<br>(5,513,655.25)
Perforated area (3D), in sq m	9,544,217.71	7,391,920.71	2,152,297.00<br>(2,411,526.25)
Core area (3D), in sq m	97,069,340.70	114,269,340.70	-17,200,000.00<br>(16,210,740.90)
Cumulative # forest fires 2000-2010 (40% forest)	9.51	16.60	-7.09<br>(7.53)

Significance levels: ***-1%, **-5%, *-10%
