# Supplementary material for: Social and Environmental Impacts of Forest Management Certification in Indonesia
Source: PLoS One. 2015 Jul 1;10(7):e0129675. doi: 10.1371/journal.pone.0129675 (PMC4488465; doi:10.1371/journal.pone.0129675)
Supplement: S5 Table — Standard errors are given in parentheses and the t-statistics-in brackets. Similar to previous results, the outcome variable for each of the treatment arms is defined as (outcome 2000-outcome 2006)-(outcome2006-outcome end year), where the end year is either 2008 or 2010. A positive value indicates that FSC increased forest cover in the treated villages relative to observationally similar non-FSC villages. (PDF) [file pone.0129675.s008.pdf]

Outcome	Treated	Controls	Bias adj. ATT
Mean %forest cover 2000-2008 (3D)	17.53	12.65	4.88<br>(3.41)<br>[1.43]
Median %forest cover 2000-2008 (3D)	17.54	8.79	8.75**<br>(3.68)<br>[2.38]
Mean %forest cover 2000-2010 (3D)	31.74	23.93	7.81**<br>(3.73)<br>[2.10]
Median %forest cover 2000-2010 (3D)	31.97	16.77	15.20***<br>(4.21)<br>[3.61]

Significance levels: ***-1%, **-5%, *-10%
