# Supplementary material for: Social and Environmental Impacts of Forest Management Certification in Indonesia
Source: PLoS One. 2015 Jul 1;10(7):e0129675. doi: 10.1371/journal.pone.0129675 (PMC4488465; doi:10.1371/journal.pone.0129675)
Supplement: S6 Table — (PDF) [file pone.0129675.s009.pdf]

Province	Concessionaire	Area (ha)	X Coordinate (decimal degrees)	Y Coordinate (decimal degrees)	Type (as of 2008)
Kalimantan Timur	PT. Intraca Wood Ind	194249.53	116.848	3.252	FSC
Kalimantan Tengah	PT. Sari Bumi Kusuma (Unit I Dan Ii)	144728.71	112.209	-0.881	FSC
Kalimantan Timur	PT. Sumalindo Lestari Jaya Ii	257792.66	115.230	1.323	FSC
Kalimantan Tengah	PT. Erna Juliawati	180489.25	111.872	-1.096	FSC
Kalimantan Timur	PT.Karya Jaya Parakawan	37404.11	117.314	3.859	Non-FSC
Kalimantan Timur	PT.Adimitra Lestari	50940.95	116.987	4.298	Non-FSC
Kalimantan Timur	PT.Sylvia Ery Timber	53790.47	117.248	3.761	Non-FSC
Kalimantan Timur	PT.INHUTANI II(SUB UNIT Malinau)	47143.08	116.541	3.063	Non-FSC
Kalimantan Timur	PT.Inhutani I (Unit Pimping)	43574.85	117.125	2.963	Non-FSC
Kalimantan Timur	PT.Meranti Sakti Indonesia II	40222.29	116.396	2.904	Non-FSC
Kalimantan Timur	PT.Sarana Trirasa Bhakti	37327.60	115.864	2.640	Non-FSC
Kalimantan Timur	PT.Sarana Trirasa Bhakti	3589.45	116.038	2.778	Non-FSC
Kalimantan Timur	PT.Inhutani I (Unit Pangean)	51115.33	116.417	2.577	Non-FSC
Kalimantan Timur	PT.Civika Wana Lestari (Eks PT. Damukti)	75361.42	116.154	2.567	Non-FSC
Kalimantan Timur	PT.Inhutani I (Unit Sambarata)	98773.09	117.223	2.507	Non-FSC
Kalimantan Timur	PT.Inhutani I (Unit Segah Hulu)	55678.04	116.902	2.473	Non-FSC
Kalimantan Timur	PT.Widya Artha Perdana	8232.50	117.726	2.363	Non-FSC
Kalimantan Timur	PT.Puji Sempurna Raharja	43293.38	117.831	2.265	Non-FSC
Kalimantan Timur	PT.Aditya Kirana Mandiri	38075.56	116.694	1.952	Non-FSC
Kalimantan Timur	PT.Amindo Wana Persada	49391.59	116.517	1.877	Non-FSC
Kalimantan Timur	PT.Wana Bhakti Persada U.	47949.20	116.715	1.823	Non-FSC
Kalimantan Timur	PT.Mardhika Insan Mulia	59018.66	117.025	1.767	Non-FSC
Kalimantan Timur	PT.Utama Damai Indah Tbr	3159.34	117.336	1.797	Non-FSC

Kalimantan Timur	PT.Mardhika Insan Mulia (Unit Tabalar)	26252.40	117.716	1.715	Non-FSC
Kalimantan Timur	PT.Karya Lestari	48166.04	116.828	1.703	Non-FSC
Kalimantan Timur	PT.Utama Damai Indah Tbr	38203.26	117.306	1.614	Non-FSC
Kalimantan Timur	PT.Widya Artha Perdana	5411.91	117.837	1.662	Non-FSC
Kalimantan Timur	PT.Segara Indochem & PT. Segara Timber	31454.40	117.604	1.593	Non-FSC
Kalimantan Timur	PT.Gunung Gajah Abadi	80086.69	116.848	1.459	Non-FSC
Kalimantan Timur	PT.Utama Damai Indah Tbr	9549.99	116.975	1.543	Non-FSC
Kalimantan Timur	PT.Segara Indochem & PT. Segara Timber	4880.61	117.750	1.541	Non-FSC
Kalimantan Timur	PT.Segara Indochem & PT. Segara Timber	48497.10	117.358	1.229	Non-FSC
Kalimantan Timur	PT.Daisy Timber	12650.61	118.499	1.318	Non-FSC
Kalimantan Timur	PT.Oceanias Timber	67729.80	116.528	1.107	Non-FSC
Kalimantan Timur	PT.Daisy Timber	33390.79	118.681	1.154	Non-FSC
Kalimantan Timur	PT.Sumber Mas Timber	55225.78	116.371	0.985	Non-FSC
Kalimantan Timur	PT.Wana Rimba Kencana	70301.73	115.841	0.999	Non-FSC
Kalimantan Timur	PT.Panambangan	43424.53	117.346	0.981	Non-FSC
Kalimantan Timur	PT.Mugitriman International	4257.33	116.310	1.046	Non-FSC
Kalimantan Timur	PT.Rodamas Tbr Kalimantan	18269.57	114.985	0.991	Non-FSC
Kalimantan Timur	PT.Sima Agung	45991.82	118.851	0.935	Non-FSC
Kalimantan Timur	PT.Mugitriman International	2466.80	116.314	0.993	Non-FSC
Kalimantan Timur	PT.Rodamas Tbr Kalimantan	18268.39	114.505	0.884	Non-FSC
Kalimantan Timur	PT.Belayan River Timber	96855.78	115.904	0.766	Non-FSC
Kalimantan Timur	PT.Marimun Timber Industri	40286.64	115.360	0.747	Non-FSC
Kalimantan Timur	PT.Melapi Timber	82382.76	116.226	0.657	Non-FSC
Kalimantan Timur	KUD.Beringin Mulya	21583.00	115.774	0.642	Non-

					FSC
Kalimantan Timur	PT.Jaya Timber Trading	18715.83	115.669	0.366	Non-FSC
Kalimantan Timur	CV.Pari Jaya Makmur	12214.80	115.562	0.447	Non-FSC
Kalimantan Timur	PT.Seroja Universum Narwastu	35729.55	115.204	0.325	Non-FSC
Kalimantan Timur	Kopontren Darussalam	21178.37	115.675	0.190	Non-FSC
Kalimantan Timur	PT.Agro City Kaltim	16463.34	114.978	0.014	Non-FSC
Kalimantan Timur	PT.Marimun Timber Industri	33210.20	116.006	-0.113	Non-FSC
Kalimantan Timur	PT.Harapan Kaltim Lestari	44683.74	115.366	-0.460	Non-FSC
Kalimantan Timur	PT.Rimba Karya Rayatama	40575.22	115.436	-0.361	Non-FSC
Kalimantan Timur	PT.Balikpapan Forest Ind.	152934.96	116.290	-1.012	Non-FSC
Kalimantan Timur	PT.Indowana Arga Timber	38849.42	115.990	-1.089	Non-FSC
Kalimantan Timur	PT.Telagamas Kalimantan	84872.59	115.876	-1.465	Non-FSC
Kalimantan Timur	PT.Telagamas Kalimantan	32576.56	115.997	-1.683	Non-FSC
Kalimantan Timur	PT.Wana Adiprima Mandiri	0.00	116.283	3.640	Non-FSC
Kalimantan Timur	PT.Essam Timber	4.27	115.918	1.656	Non-FSC
Kalimantan Timur	PT.Essam Timber	933.37	116.020	1.799	Non-FSC
Kalimantan Timur	PT.Mugitriman International	87.01	115.916	1.635	Non-FSC
Kalimantan Timur	PT.Mugitriman International	796.21	116.303	1.449	Non-FSC
Kalimantan Timur	PT.Mugitriman International	2998.17	116.317	1.323	Non-FSC
Kalimantan Timur	PT.Mugitriman International	0.00	116.321	1.262	Non-FSC
Kalimantan Timur	PT.Mugitriman International	24.13	116.043	1.117	Non-FSC
Kalimantan Timur	PT.Mugitriman International	588.48	116.031	1.165	Non-FSC
Kalimantan Timur	PT.Mugitriman International	672.62	116.323	1.287	Non-FSC
Kalimantan Timur	PT.Mugitriman International	2329.46	116.292	1.649	Non-FSC

Kalimantan Timur	PT.Mugitriman International	284.25	116.275	1.639	Non-FSC
Kalimantan Timur	PT.Mugitriman International	228.66	116.322	1.665	Non-FSC
Kalimantan Timur	PT.Rodamas Tbr Kalimantan	24737.16	114.799	0.846	Non-FSC
Kalimantan Timur	PT.Triwira Asta Barata	62.66	115.594	0.058	Non-FSC
Kalimantan Timur	PT.Timber Dana	23.65	115.739	-0.950	Non-FSC
Kalimantan Timur	PT.Rodamas Tbr Kalimantan	53774.94	115.798	-0.737	Non-FSC
Kalimantan Timur	PT.Wana Adiprima Mandiri	33116.55	116.409	3.611	Non-FSC
Kalimantan Timur	PT.Permata Borneo Abadi	32630.60	116.213	3.509	Non-FSC
Kalimantan Timur	PT.Essam Timber	328836.29	115.741	1.894	Non-FSC
Kalimantan Timur	PT.Mugitriman International	136.43	116.290	1.623	Non-FSC
Kalimantan Timur	PT.Mugitriman International	45.42	116.319	1.680	Non-FSC
Kalimantan Timur	PT.Karya Wijaya Sukses	7.71	114.776	0.855	Non-FSC
Kalimantan Timur	PT.Rimba Sempana Makmur	27445.78	114.524	0.714	Non-FSC
Kalimantan Timur	PT.Triwira Asta Barata	63291.12	115.775	0.074	Non-FSC
Kalimantan Timur	PT.Triwira Asta Barata	0.00	115.573	0.048	Non-FSC
Kalimantan Timur	PT.Timber Dana	77598.57	115.618	-0.923	Non-FSC
Kalimantan Timur	PT.Batu Karang Sakti	47580.84	116.140	3.397	Non-FSC
Kalimantan Timur	PT.Karya Wijaya Sukses	22529.13	114.784	0.790	Non-FSC
Kalimantan Timur	PT.Inhutani I (Unit Kunyit - Simendurut)	68536.65	116.695	4.085	Non-FSC
Kalimantan Timur	PT.Inhutani I (Unit Kunyit - Simendurut)	52152.24	116.536	3.877	Non-FSC
Kalimantan Timur	PT.Inhutani I (Unit Meraang)	148907.79	117.087	2.021	Non-FSC
Kalimantan Timur	PT.Inhutani I (Unit Labanan)	72389.46	117.457	1.835	Non-FSC
Kalimantan Timur	PT.Mugitriman International	174092.99	116.044	1.430	Non-FSC
Kalimantan Timur	PT.Hanurata Coy Ltd Unit	38292.57	118.109	1.192	Non-

	Sangkulirang (62400 ha) - Unit Berau (79200 Ha)				FSC
Kalimantan Timur	PT.Nadila Indodaya	34283.66	118.476	1.001	Non-FSC
Kalimantan Timur	PT.Nadila Indodaya	12038.10	118.119	1.099	Non-FSC
Kalimantan Timur	PT.Hanurata Coy Ltd Unit Sangkulirang (62400 ha) - Unit Berau (79200 Ha)	91546.82	118.600	1.054	Non-FSC
Kalimantan Timur	PT.Rizki Kacida Reana	29395.61	115.698	-1.517	Non-FSC
Kalimantan Timur	PT.Jaya Timber Trading	35954.78	115.836	0.499	Non-FSC
Kalimantan Timur	PT.Greaty Sukes Abadi	13241.31	116.449	-1.270	Non-FSC
Kalimantan Timur	PT.Greaty Sukes Abadi	19127.83	116.271	-1.183	Non-FSC
Kalimantan Timur	KSU.Meranti Tumbuh Indah	16544.25	116.744	4.208	Non-FSC
Kalimantan Timur	PT.ITCI Kayan Hutani (PT. IKANI)	132598.01	116.867	2.887	Non-FSC
Kalimantan Timur	PT.ITCI Kayan Hutani (PT. IKANI)	95624.89	117.049	2.658	Non-FSC
Kalimantan Timur	PT.Kedungmadu Tropical Wood	66862.88	117.456	1.480	Non-FSC
Kalimantan Timur	PT.Intertropic Aditama	45885.77	116.549	1.251	Non-FSC
Kalimantan Timur	PT.Kiani Lestari (eks PT GPI)	216398.62	116.992	0.878	Non-FSC
Kalimantan Timur	PT.Barito Nusantara Indah	101200.26	115.535	0.951	Non-FSC
Kalimantan Timur	PT.Limbang Ganesa	107107.20	115.988	0.268	Non-FSC
Kalimantan Timur	PT.Ratah Timber	3231.52	115.171	0.252	Non-FSC
Kalimantan Timur	PT.Ratah Timber	8335.10	115.053	0.201	Non-FSC
Kalimantan Timur	PT.I T C I/ITCIKU	263282.38	116.488	-0.708	Non-FSC
Kalimantan Timur	PT.Mutiara Kalja Permai	3554.89	116.025	1.235	Non-FSC
Kalimantan Timur	PT.Ratah Timber	785.38	115.364	0.097	Non-FSC
Kalimantan Timur	PT.Ratah Timber	2.30	115.413	0.130	Non-FSC
Kalimantan Timur	PT.Narkata Rimba	69590.76	116.480	1.491	Non-FSC

Kalimantan Timur	PT.Mutiara Kalja Permai	54782.07	115.954	1.031	Non-FSC
Kalimantan Timur	PT.Ratah Timber	76843.64	115.196	0.098	Non-FSC
Kalimantan Timur	PT.Kemakmuran Berkah Timber	112337.27	114.761	1.028	Non-FSC
Kalimantan Timur	PT.Borneo Karya Indah Mandiri	48352.29	118.290	1.060	Non-FSC
Kalimantan Timur	PT.Sumalindo Lestari Jaya IV (eks PT. Madyakara Pacific)	102518.96	116.767	2.227	Non-FSC
Kalimantan Timur	PT.Rizki Kacida Reana	17993.48	117.391	2.482	Non-FSC
Kalimantan Timur	PT.Rizki Kacida Reana	37482.17	117.802	2.415	Non-FSC
Kalimantan Tengah	PT.Mitra Perdana Palangka	0.00	114.074	0.423	Non-FSC
Kalimantan Tengah	PT.Sarang Sapta Putra	51011.32	114.326	0.229	Non-FSC
Kalimantan Tengah	PT.Kayu Ara Jaya Raya	88062.16	113.811	-0.015	Non-FSC
Kalimantan Tengah	PT.Fortuna Cipta Sejahtera	23865.01	114.470	0.277	Non-FSC
Kalimantan Tengah	PT.Fortuna Cipta Sejahtera	18992.40	114.786	-0.163	Non-FSC
Kalimantan Tengah	PT.Fortuna Cipta Sejahtera	11911.90	115.111	-0.263	Non-FSC
Kalimantan Tengah	PT.Kayu Ara Jaya Raya	6.96	113.604	-0.075	Non-FSC
Kalimantan Tengah	PT.Pemantang Abaditama	54737.62	114.676	-0.228	Non-FSC
Kalimantan Tengah	Kop.Putra Dayak Jaya	24748.06	113.631	-0.210	Non-FSC
Kalimantan Tengah	Kop.Putra Dayak Jaya	31.71	113.716	-0.121	Non-FSC
Kalimantan Tengah	Kop.Putra Dayak Jaya	260.57	113.601	-0.182	Non-FSC
Kalimantan Tengah	PT.Kahayan Terang Abadi	39169.01	113.749	-0.429	Non-FSC
Kalimantan Tengah	Kop.Putra Dayak Jaya	1.70	113.713	-0.246	Non-FSC
Kalimantan Tengah	PT.Kahayan Terang Abadi	13.15	113.708	-0.246	Non-FSC
Kalimantan Tengah	Kop.Putra Dayak Jaya	1.72	113.707	-0.249	Non-FSC
Kalimantan Tengah	PT.Kahayan Terang Abadi	4.40	113.704	-0.250	Non-FSC
Kalimantan Tengah	PT.Barito Putera	27632.12	114.583	-0.338	Non-

					FSC
Kalimantan Tengah	PT.Kahayan Terang Abadi	1312.96	113.715	-0.287	Non-FSC
Kalimantan Tengah	PT.Wana Inti Kahuripan Intiga	92014.61	115.105	-0.456	Non-FSC
Kalimantan Tengah	PT.Hutan Domas Raya	110075.34	113.534	-0.531	Non-FSC
Kalimantan Tengah	PT.Carus Indonesia	72121.99	113.242	-0.633	Non-FSC
Kalimantan Tengah	PT.Praba Nugraha Tech.	41530.04	113.832	-0.684	Non-FSC
Kalimantan Tengah	PT.Austral Byna	267319.64	115.279	-0.970	Non-FSC
Kalimantan Tengah	PT.Barito Putera	14527.80	115.334	-0.696	Non-FSC
Kalimantan Tengah	PT.Sarana Piranti Utama	52272.73	112.862	-0.737	Non-FSC
Kalimantan Tengah	PT.Gaung Satya Graha Agrindo	49708.67	113.010	-0.832	Non-FSC
Kalimantan Tengah	PT.Fitamaya Asmapara	43607.71	113.146	-0.809	Non-FSC
Kalimantan Tengah	PT.Sikatan Wana Raya	48690.38	113.450	-0.767	Non-FSC
Kalimantan Tengah	PT.Pandu Jaya Gemilang Agung	46871.79	114.317	-0.871	Non-FSC
Kalimantan Tengah	PT.Graha Sentosa Permai	45261.95	112.844	-0.929	Non-FSC
Kalimantan Tengah	PT.Dwima Jaya Utama	71024.71	113.210	-1.025	Non-FSC
Kalimantan Tengah	PT.Dasa Intiga	131427.19	114.490	-1.253	Non-FSC
Kalimantan Tengah	PT.Dwima Jaya Utama	240.22	113.259	-0.891	Non-FSC
Kalimantan Tengah	PT.Meranti Mustika	58831.91	112.274	-1.082	Non-FSC
Kalimantan Tengah	Kop.Mandau Talawang	47328.37	114.065	-1.053	Non-FSC
Kalimantan Tengah	PT.Dwima Jaya Utama	57104.21	112.858	-1.092	Non-FSC
Kalimantan Tengah	PT.Hasil Kalimantan Jaya	51519.32	113.596	-1.309	Non-FSC
Kalimantan Tengah	PT.Yakin Timber Jaya	28967.11	112.571	-1.107	Non-FSC
Kalimantan Tengah	PT.Hutanindo Lestari Raya Timber	97116.94	111.648	-1.378	Non-FSC
Kalimantan Tengah	PT.Sinergi Hutan Sejati	67136.08	114.693	-1.337	Non-FSC

Kalimantan Tengah	PT.Indexim Utama Corp.	51074.12	115.608	-1.253	Non-FSC
Kalimantan Tengah	PT.Sindo Lumber	90065.08	115.245	-1.366	Non-FSC
Kalimantan Tengah	PT.Hutan Mulya	48368.82	112.895	-1.266	Non-FSC
Kalimantan Tengah	PT.Sarmiento Parakanca Tbr	214245.76	112.122	-1.538	Non-FSC
Kalimantan Tengah	PT.Kayu Tribuana Rama	113906.13	112.443	-1.366	Non-FSC
Kalimantan Tengah	PT.Anugrah Alam Barito	39356.83	114.098	-1.330	Non-FSC
Kalimantan Tengah	PT.Trisetia Citagraha	27120.89	115.320	-1.485	Non-FSC
Kalimantan Tengah	PT.Central Kalimantan Abadi	40650.12	111.924	-1.604	Non-FSC
Kalimantan Tengah	PT.Karda Trades	91691.40	111.448	-1.558	Non-FSC
Kalimantan Tengah	PT.Erythrina Nugraha Megah	41993.48	111.799	-1.549	Non-FSC
Kalimantan Tengah	PT.Berkat Cahaya Timber	145309.51	112.402	-1.622	Non-FSC
Kalimantan Tengah	PT. Sari Bumi Kusuma (Kalteng2)	60386.08	111.071	-1.696	Non-FSC
Kalimantan Tengah	PT.Tingang Karya Mandiri	42303.20	114.696	-1.567	Non-FSC
Kalimantan Tengah	PT.Amprah Mitra Jaya	79426.62	111.231	-1.725	Non-FSC
Kalimantan Tengah	PT.Hasnur Jaya Utama	33002.02	115.263	-1.645	Non-FSC
Kalimantan Tengah	PT.Trisetia Intiga	78737.13	111.630	-1.767	Non-FSC
Kalimantan Tengah	PT.Intrado Jaya Intiga	46598.55	111.864	-1.764	Non-FSC
Kalimantan Tengah	PT.Trisetia Intiga	19653.19	111.501	-1.969	Non-FSC
Kalimantan Tengah	PT.Intrado Jaya Intiga	0.04	112.052	-1.902	Non-FSC
Kalimantan Tengah	PT.Intrado Jaya Intiga	5160.20	112.052	-1.946	Non-FSC
Kalimantan Tengah	PT.Dasa Intiga	52322.04	111.194	-2.938	Non-FSC
Kalimantan Tengah	PT.Mitra Perdana Palangka	56523.34	113.924	0.268	Non-FSC
Kalimantan Tengah	PT.Menorah Loggingindo	56079.25	114.409	0.047	Non-FSC
Kalimantan Tengah	PT.Karya Delta Permai	79072.69	114.290	-0.276	Non-

					FSC
Kalimantan Tengah	PT.Gunung Meranti	90103.62	113.906	-0.464	Non-FSC
Kalimantan Tengah	PT.Kayu Waja	39350.52	112.577	-1.003	Non-FSC
Kalimantan Tengah	PT.Berkat Cahaya Timber	20125.28	112.324	-1.192	Non-FSC
Kalimantan Tengah	PT.Rinanda Inti Lestari	41510.40	113.169	-1.275	Non-FSC
Kalimantan Tengah	PT.Bina Multi Alam Lestari	34876.61	114.599	-0.924	Non-FSC
Kalimantan Tengah	PT.Taman Raja Persada	58251.28	114.496	-0.097	Non-FSC
Kalimantan Tengah	PT.Akhates Plywood	94527.62	114.743	0.332	Non-FSC
Kalimantan Tengah	PT.Lestari Damai Indah Tbr	10965.81	115.187	-0.243	Non-FSC
Kalimantan Selatan	PT.Elbana Abadi Jaya	4214.73	115.676	-1.941	Non-FSC
Kalimantan Selatan	PT.Aya Yayang Indonesia	91608.09	115.478	-1.625	Non-FSC
Kalimantan Selatan	PT.Elbana Abadi Jaya	11057.87	115.637	-1.604	Non-FSC
Kalimantan Barat	PT.Sinergi Bumi Lestari	13495.41	111.357	-0.823	Non-FSC
Kalimantan Barat	PT.Suka Jaya Makmur	149009.03	111.172	-1.232	Non-FSC
Kalimantan Barat	PT.Suka Jaya Makmur	42993.44	110.819	-1.493	Non-FSC
Kalimantan Barat	PT.Harapan Kita Utama	35088.60	112.691	0.107	Non-FSC
Kalimantan Barat	PT.Batasan (Kalbar)	16329.14	112.810	0.125	Non-FSC
Kalimantan Barat	CV.Bakti Dwipa Kariza	11987.87	112.185	0.259	Non-FSC
Kalimantan Barat	PT.Harapan Kita Utama	5120.77	112.486	0.248	Non-FSC
Kalimantan Barat	PT.Karya Rekanan Binabersama	45123.46	112.257	0.127	Non-FSC
Kalimantan Barat	PT.Sari Bumi Kusuma (Kalbar)	48071.70	113.010	-0.275	Non-FSC
Kalimantan Barat	PT.Batasan (Kalbar)	8969.14	113.134	-0.381	Non-FSC
Kalimantan Barat	PT.Sari Bumi Kusuma (Kalbar)	12166.04	113.108	-0.420	Non-FSC
Kalimantan Barat	PT.Sari Bumi Kusuma (Kalbar)	5198.89	112.708	-0.457	Non-FSC

Kalimantan Barat	PT.Batasan (Kalbar)	454.04	112.829	-0.452	Non-FSC
Kalimantan Barat	PT.Batasan (Kalbar)	12305.67	112.734	-0.492	Non-FSC
Kalimantan Barat	PT.Kalimantan Satya Kencana	27807.32	111.876	-0.748	Non-FSC
Kalimantan Barat	PT.Mohairson Pawan Khatulistiwa	48222.99	110.197	-1.612	Non-FSC
Kalimantan Barat	PT.Kawedar Wood Industry	58883.53	113.309	0.459	Non-FSC
Kalimantan Barat	PT.Kawedar Wood Industry	9748.19	113.070	0.745	Non-FSC
Kalimantan Barat	PT.Duaja Corp. II	71369.07	110.761	-0.770	Non-FSC
Kalimantan Barat	PT.Wanasokan Hasilindo	46824.72	111.204	-0.980	Non-FSC
Kalimantan Barat	PT.Bina Ovivipari Semesta	6115.99	109.401	-0.850	Non-FSC
Kalimantan Barat	PT.Bina Ovivipari Semesta	1416.50	109.409	-0.887	Non-FSC
Kalimantan Barat	PT.Bina Ovivipari Semesta	4062.02	109.652	-0.947	Non-FSC
Kalimantan Barat	PT.Bina Ovivipari Semesta	11.71	109.646	-0.965	Non-FSC
Kalimantan Barat	PT.Wana Kayu Batu Putih	43103.28	110.954	-1.922	Non-FSC
Kalimantan Barat	PT.Sewaka Lahan Sentosa	17817.81	111.030	-0.831	Non-FSC
Kalimantan Barat	PT.Sewaka Lahan Sentosa	7559.29	110.883	-1.337	Non-FSC
Kalimantan Barat	PT.Sewaka Lahan Sentosa	6998.57	110.877	-1.131	Non-FSC
Kalimantan Barat	PT.Karya Rekanan Binabersama	80.01	112.047	0.098	Non-FSC
Kalimantan Barat	PT.Batasan (Kalbar)	2892.18	112.560	-0.538	Non-FSC
Kalimantan Barat	PT.Karunia Hutan Lestari	40494.16	110.836	-0.889	Non-FSC
Kalimantan Barat	CV.Pangkar Begili	30286.37	112.397	-0.348	Non-FSC
Kalimantan Barat	PT.Kandelia Alam	18031.94	109.510	-0.605	Non-FSC
